# Inhibition of FABP6 Reduces Tumor Cell Invasion and Angiogenesis through the Decrease in MMP-2 and VEGF in Human Glioblastoma Cells

**DOI:** 10.3390/cells10102782

**Published:** 2021-10-17

**Authors:** Feng-Cheng Pai, Hsiang-Wei Huang, Yu-Ling Tsai, Wen-Chiuan Tsai, Yu-Chen Cheng, Hsin-Han Chang, Ying Chen

**Affiliations:** 1 Department of Emergency Medicine, Tri-Service General Hospital, National Defense Medical Center, Taipei 11490, Taiwan; ssbb15@gmail.com; 2 Department of Biology and Anatomy, National Defense Medical Center, Taipei 11490, Taiwan; m860502@yahoo.com.tw (H.-W.H.); plokmijzz@gmail.com (Y.-C.C.); 3 Department of Pathology, Tri-Service General Hospital, National Defense Medical Center, Taipei 11490, Taiwan; c909228@gmail.com (Y.-L.T.); ab95057@hotmail.com (W.-C.T.)

**Keywords:** FABP6, invasion, angiogenesis, glioblastoma

## Abstract

Malignant glioma is one of the most lethal cancers with rapid progression, high recurrence, and poor prognosis in the central nervous system. Fatty acid-binding protein 6 (FABP6) is a bile acid carrier protein that is overexpressed in colorectal cancer. This study aimed to assess the involvement of FABP6 expression in the progression of malignant glioma. Immunohistochemical analysis revealed that FABP6 expression was higher in glioma than in normal brain tissue. After the knockdown of FABP6, a decrease in the migration and invasion abilities of glioma cells was observed. The phosphorylation of the myosin light chain was inhibited, which may be associated with migration ability. Moreover, expression levels of invasion-related proteins, matrix metalloproteinase-2 (MMP-2) and cathepsin B, were reduced. Furthermore, tube formation was inhibited in the human umbilical vein endothelial cells with a decreased concentration of vascular endothelial growth factor (VEGF) in the conditioned medium after the knockdown of FABP6. The phosphorylation of the extracellular signal-regulated kinase (ERK), c-Jun NH2-terminal kinase (JNK), and p65 were also decreased after FABP6 reduction. Finally, the bioluminescent images and immunostaining of MMP-2, cluster of differentiation 31 (CD31), and the VEGF receptor 1 (VEGFR1) revealed attenuated tumor progression in the combination of the FABP6-knocked-down and temozolomide (TMZ)-treated group in an orthotopic xenograft mouse tumor model. This is the first study that revealed the impact of FABP6 on the invasion, angiogenesis, and progression of glioma. The results of this study show that FABP6 may be a potential therapeutic target combined with TMZ for malignant gliomas.

## 1. Introduction

Gliomas account for the majority of primary tumors that arise within the brain parenchyma and are the most common intracranial neoplasms [1]. According to the 2016 edition of the World Health Organization’s (WHO) revised classification, high-grade gliomas include glioblastoma, anaplastic astrocytoma, and anaplastic oligodendroglioma [2]. The initial treatment for high-grade gliomas is surgical resection, if accessible, combined with adjuvant post-operative temozolomide (TMZ)-based chemoradiotherapy. However, due to the infiltration of tumor cells, complete resection and adjuvant therapy are elusive, resulting in a higher percentage of recurrence and worse prognosis in patients with high-grade gliomas than in patients with low-grade gliomas [3].

Fatty acid-binding proteins (FABPs) modulate the metabolism of fatty acids, cell growth, and proliferation. In 2000, Jing et al. revealed that the dysregulation of FABP plays important roles in carcinogenesis as well as the progression and metastasis of cancer [4]. In carcinoma cell lines, distinct differences were observed in the FABP expression patterns of cells derived from different tumors [5]. FABPs affect tumor progression via specific pathways. In hepatocellular carcinoma (HCC), the upregulated fatty acid-binding protein 1 (FABP1) interacts with the VEGF receptor and Src via the focal adhesion kinase (FAK) and enhances the expression of the angiogenic vascular endothelial growth factor A (VEGF-A) [6]. In addition, FABP4 promotes tumor progression by altering the activities of matrix metalloproteinases (MMPs), especially MMP-2 and MMP-9, in prostate cancer [7]. FABP4 is also associated with the mechanistic target of rapamycin complex 1 (mTORC1) in the human umbilical vein endothelial cells (HUVECs) as well as pro-angiogenic signals including mitogen-activated protein kinase (MAPK), endothelial nitric oxide synthase (eNOS), and stem cell factor (SCF)/c-kit [8]. Accordingly, tumor cells can obtain more oxygen and nutrients and clear waste products, which leads to the growth, progression, and metastasis of cancer.

FABP6, also known as gastrotropin or ileal FABP, can transport bile acids and is highly expressed in the ileum. FABP6 has a higher affinity for bile acids than fatty acids. Previous studies have revealed a link between bile acids, FABP6, and colorectal carcinogenesis in animal models [9]; however, whether the effect of FABP6 on glioma remains unknown. In the central nervous system, fatty acids and FABPs impact the growth and function of the brain [10]. This implies that lipid dysregulation may play a role in glioma development [11]. Accordingly, this is the first study to investigate the role of FABP6 in the progression of glioma.

## 2. Materials and Methods

### 2.1. Immunohistochemical Staining of Human Glioma Specimens

A glioma tissue microarray (GL807a; US Biomax Inc, Derwood, MD, USA) was incubated in 5% non-fat milk and with a rabbit anti-human FABP6 monoclonal antibody (Abcam) at 4 °C overnight. After 16 h of incubation, the specimens were incubated with biotin-labeled secondary immunoglobulin and 3-amino-9-ethylcarbazole substrate chromogen (DAKO, Glostrup, Denmark) to observe peroxidase activity.

### 2.2. Cell Culture

Two human glioma tumor cell lines, LN229 and U87MG, were obtained from American Type Culture Collection (ATCC). The glioma cells were cultured in Roswell Park Memorial Institute (RPMI) 1640 medium supplemented with 10% fetal bovine serum (FBS). HUVECs were obtained from the Bioresource Collection and Research Center in Taiwan and cultured with an endothelial cell medium (ScienCell Research Laboratories, Carlsbad CA, USA). All cells were cultured in an incubator at 37 °C with 5% carbon dioxide (CO_2_).

### 2.3. Antibodies

The information on antibodies is shown in Table 1.

### 2.4. Stable Expression of shRNAs

The shRNA clones (TRCN0000059723, TRCN0000059724, TRCN00000419834, and TRCN00000447012) and lentiviral package vectors (pCMV-dR8.91 and pMD2.G) were obtained by the National RNAi Core Facility at Academia Sinica in Taiwan. The LN229 and U87MG glioma cells were infected with the virus and incubated with puromycin to select the stably infected cells.

### 2.5. Real-Time Polymerase Chain Reaction (RT-PCR)

Cells were seeded in 12-well plates and treated with a transfection reagent. After 72 h, the culture medium was removed from the plates. Following the cells were lysed by GENEzol™ Reagent, and purified the total RNA by the GENEzol™ TriRNA Pure Kit from Geneaid in Taiwan. Up to 500 ng of total RNA could be reverse transcribed in the reaction mixture, and synthesis of cDNA template was performed using the PrimeScript™ RT reagent Kit (TAKARA Bio Inc, Shiga, Japan) according to the manufacturer’s instructions. Real time RT-PCR was carried out by LightCycler^®^ 480 Instrument (Roche, Basel, Swiss) using SensiFAST SYBR (Meridian Bioscience, Cincinnati, OH, USA).

### 2.6. 3-(4,5-Dimethylthiazol-2-yl)-2,5-diphenyltetrazolium Bromide (MTT) Assay

Both 5 × 103 shScramble and shFABP6 stably expressing LN229 and U87MG glioma cells were cultured in a 96-well plate. The MTT assay was performed after 24, 48, and 72 h to determine the viabilities of cells. The absorbance was measured at 590 nm after 4 h of MTT application.

### 2.7. Cell Migration and Invasion Assays

Wound healing and transwell assays were performed to assess the migration abilities of tumor cells. In the wound healing assay, LN229 and U87MG glioblastoma cells were seeded in a 6-well plate. Thereafter, a P200 pipette tip was used to scrape cells for imaging after 16 h. The wound area was analyzed using ImageJ software. The transwell migration assay was conducted by seeding 2 × 105 LN229 or U87MG glioblastoma cells in the upper chamber of a Transwell insert (Corning, Midland, NC, USA). After 16 h of incubation, the cells on the lower side were stained and examined under a microscope. The transwell invasion assay was performed by seeding 2 × 104 LN229 or U87MG cells in the upper chamber of the insert. Before seeding, Matrigel (BD, Franklin Lakes, NJ, USA) was added to the upper chamber of a medium for 24 h. After 16 h of incubation, the cells in the lower chamber were fixed and stained with the Coomassie blue dye. The invaded cells were counted in three randomly selected fields from each membrane in six independent experiments.

### 2.8. Western Blotting

The glioma cells were homogenized by a protein extraction buffer (GE Healthcare Life Sciences, Chicago, IL, USA) applied with proteinase and phosphatase inhibitors (MedChemExpress, Monmouth Jucntion, NJ, USA). Electrophoresis was performed on a 10% sodium dodecyl sulphate-polyacrylamide gel electrophoresis (SDS-PAGE) gel, and the protein samples were transferred to a nitrocellulose membrane (Bio-Rad, Berkeley, CA, USA). Strips from the membrane were incubated with 5% non-fat milk in Tris-buffered saline (pH 7.4) containing 0.1% Tween (TBS-Tween). The membranes were then incubated in blocking solution with the primary antibodies overnight at 4 °C. After washing, the strips were incubated with a 1:5000 or 1:10,000 dilution of the horseradish peroxidase (HRP)-conjugated anti-rabbit or anti-mouse immunoglobulin G (IgG) antibodies from Cell Signaling Technology. Subsequently, the blots were incubated in the developing solution with the electrochemiluminescence (ECL) substrate (Bio-Rad). The densities of the bands on the membrane were captured and quantified by the ImageJ software. The density of the control sample was set as 100% and the densities of the test samples were relative to that of the internal control. At least six independent experiments were performed.

### 2.9. Tube Formation Assay

Matrigel (50 μL/well) was added to a 96-well plate and incubated for 1 h at 37 °C before the assay. Thereafter, 1× 10^4^ HUVECs were incubated with the conditioned medium collected from the 5 × 10^5^ LN229 or U87MG cells in 6-well plates for 48 h. The number of nodes and tubes of the tubular structures after 6 h of incubation was recorded and analyzed using the AngioTool online software. VEGFA121 and VEGFA165 were obtained from Sino Biological Inc in Beijing, China.

### 2.10. Orthotopic Xenograft Animal Model

All animal experiments were approved by the experimental animal center of the National Defense Medical Center of Taiwan (IACUC No. 19-157). Nude mice with an average weight of 20–25 g were used. After administration of anesthetics, 1 × 10^5^ FABP6 knockdown (clone 724)- or shScramble control (clone 004)-Luc2 cells were implanted into the right hemisphere of mice. Five days after implantation, they were divided into the following four groups: shScramble control, shScramble control plus TMZ, FABP6 knockdown, and FABP6 knockdown group plus TMZ. Body weight was measured every three days, and the tumor size was detected by IVIS spectrum (PerkinElmer, Waltham, MA, USA). TMZ was administered orally on the 6th day lasting for 9 days. After sacrifice, the brain was dissected out and fixed with 10% formalin, embedded in paraffin, and sectioned. The samples were then stained with hematoxylin and eosin (HE).

### 2.11. Histological and Immunohistochemical Examination

The brain tissues were excised, rinsed twice in phosphate-buffered saline (PBS), and fixed in 10% formaldehyde. Thereafter, the tissues were frozen and sliced into 5 μm thick sections. Then, HE stain was applied to facilitate the histological evaluation. The expression levels of MMP2, cluster of differentiation 31 (CD31), and VEGFR1 were detected in the brain tumors of nude mice by immunohistochemical (IHC) staining. The IHC staining was conducted by Ventana BenchMark ULTRA system (Roche, Basel, Swiss). The primary antibody was diluted in Antibody Dilution Buffer (Ventana). The antigen retrieval was performed according to the manufacturer’s standard protocol. The secondary goat anti-rabbit antibodies (Jackson ImmunoResearch Laboratories, West Grove, PA, USA) were used. The aforementioned protein expression was observed in 10 random fields in each group.

### 2.12. Terminal Deoxynucleotidyl Transferase (TdT) dUTP Nick-End Labeling (TUNEL) Assay

The TUNEL staining was performed according to the manufacturer (In Situ Cell Death Detection Kit, Fluorescein (Roche, Basel, Swiss). The pictures were photographed with BX51 (Olympus, Tokyo, Japan).

### 2.13. Enzyme-Linked Immunosorbent Assay (ELISA) for VEGF in Condition Medium

The concentration of VEGF-A was measured by the ELISA Kit (R&D Systems, Minneapolis, MN, USA) according to the manufacturer’s instructions. The values detected by ELISA were corrected using a dilution factor and expressed as pg/mL.

### 2.14. Statistical Analysis

The overall survival data sets obtained from The Cancer Genome Atlas (TCGA) database were analyzed using the Kaplan–Meier method on the Gene Expression Profiling Interactive Analysis (GEPIA) website. All experiments were performed at least three times, and the results are expressed as the mean ± standard error of the mean (SEM). The differences between means were assessed by the Kruskal–Wallis test. The Mann–Whitney U test was applied for post-hoc analysis. Statistical significance was estimated at *p* < 0.05.

## 3. Results

### 3.1. FABP6 Had Higher Expression in Glioma

**Figure 1:**
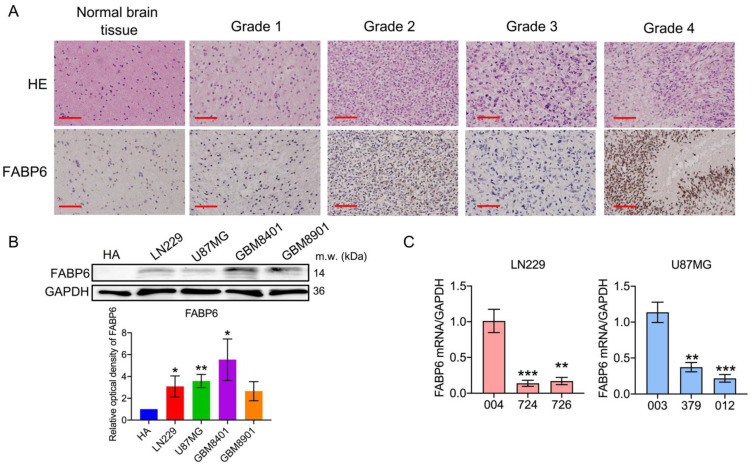
Fatty acid-binding protein 6 (FABP6) expression in human glioblastoma cells: (**A**) Hematoxylin and eosin (HE) staining (left column) of the normal brain tissue, pilocytic astrocytoma, diffuse astrocytoma, anaplastic astrocytoma, and glioblastoma multiforme. Immunohistological analysis of FABP6 in the right column (original magnification was ×400). Scale bar = 50 μm. (**B**) The expression levels of FABP6 in the glioma cell lines were analyzed by Western blotting. GAPDH was used as a loading control. * *p* < 0.05; ** *p* < 0.01 compared with the human astrocytes group. (**C**) Reverse transcription-polymerase chain reaction (RT-PCR) was used to analyze the mRNA expression of FABP6 after knockdown with shFABP6. ** *p* < 0.01; *** *p* < 0.001 compared with the shScramble control group (004 in LN229 cells and 003 in U87MG cells).

As shown in Figure 1A, the expression levels of FABP6 were noted in the normal and tumor tissues. The expression of FABP6 was found to be elevated in the neoplastic brain tissue and was not proportional to the grade of the glioma. FABP6 expression was significantly higher in the glioma cell lines (Figure 1B). The relationship between the survival and expression of FABP6 was also analyzed by GEPIA and TCGA database (Figure S1).

### 3.2. Analysis of the Expression of FABP and Establishment of shRNA (shFABP6) Stable Clones in Glioma Cell Lines

Stable clones were then established in two cell lines, LN229 and U87MG. The knockdown effects of shFABP6 in the LN229 (724 and 726) and U87MG (379 and 012) cells were confirmed by reverse transcription-polymerase chain reaction (RT-PCR) (Figure 1C). In addition, the protein expression levels of FABP6 in the LN229 and U87MG cells were found to be reduced (Figure S2A). However, cell growth was unaffected between FABP6 knockdown and scramble control groups by MTT assay (Figure S2B) and colony formation (Figure S2C,D).

### 3.3. Knockdown of FABP6 Decreased the Migration and Invasion Abilities of Invasion-Related Proteins in the LN229 and U87MG Cells

**Figure 2:**
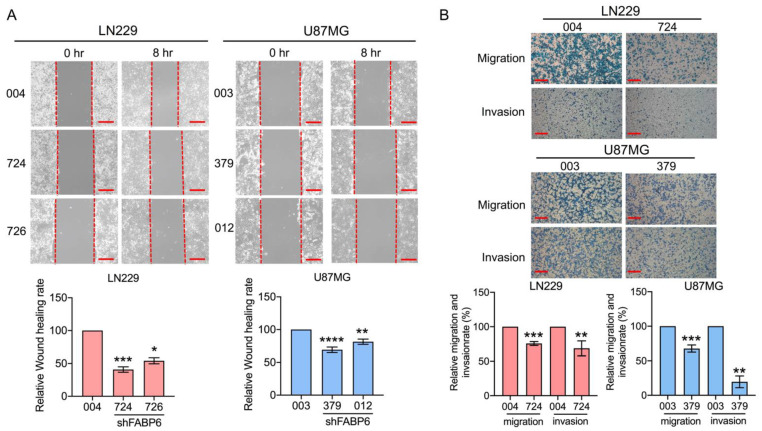
The migration and invasion abilities after the inhibition of FABP6: (**A**) Wound healing migration assays were performed in two human glioma cell lines, LN229 (*n* = 6) and U87MG (*n* = 7), after 8 h scratch. (**B**) Transwell migration assays were performed in the LN229 and U87MG cells after 16 h incubation. Thereafter, the cells were stained and captured. * *p* < 0.05; ** *p* < 0.01; *** *p* < 0.001; **** *p* < 0.0001 compared with the shScramble control group (004 in LN229 cells and 003 in U87MG cells). The lower panel displays the relative rate of the transwell migration ability compared to that of the control: 004 and 003 are the shScramble control groups in LN229 and U87MG, 724 and 726 in LN229 cells, and 379 and 012 in U87MG cells were the shFABP knockdown groups. Scale bar = 200 μm.

**Figure 3:**
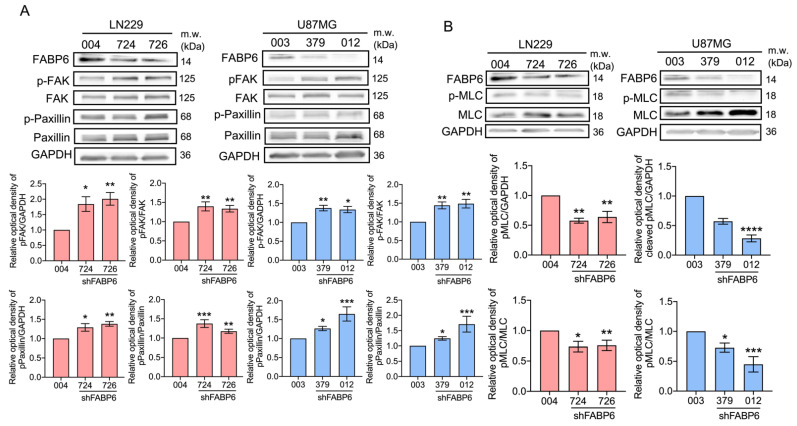
Effects of shFABP6 on the expression levels of the focal adhesion-related proteins in glioma cells: (**A**) focal adhesion kinase (FAK), phospho-focal adhesion kinase (p-FAK), paxillin, and phospho-paxillin (p-paxillin); (**B**) myosin light chain (MLC) and phospho-myosin light chain (p-MLC) were analyzed by Western blotting in the LN229 and U87MG cells using the shScramble control group (004 in LN229 cells and 003 in U87MG cells) and shFABP6. Glyceraldehyde-3-phosphate dehydrogenase (GAPDH) was used as the endogenous control. The plotted graphs show the relative quantitative analysis of the aforementioned proteins. * *p* < 0.05; ** *p* < 0.01; *** *p* < 0.001; **** *p* < 0.0001 compared with the shScramble control group.

The wound healing assays demonstrated that the cell migration abilities were reduced in glioma cells compared with those in control cells (Figure 2A). In the transwell assays, the migration and invasion abilities decreased significantly after shFABP6 knockdown (Figure 2B). Interestingly, after FABP6 inhibition, the phosphorylation of FAK and paxillin increased. Simultaneously, the expression levels of the phospho-myosin light chain (p-MLC) were significantly reduced in both LN229 and U87MG cells (Figure 3). These results suggested that the decrease in migration abilities by FABP6 knockdown may be associated with the inhibition of MLC in glioma cells.

**Figure 4:**
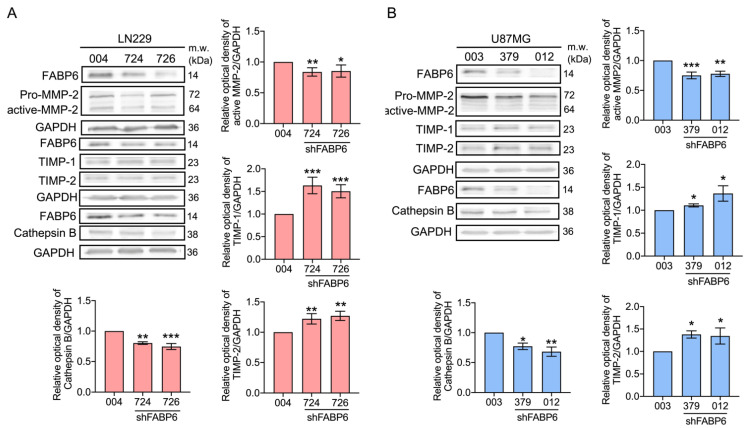
Effects of shFABP6 on the expression levels of the extracellular matrix (ECM) degradation-associated proteins in LN229 and U87MG cells. The expression levels of matrix metalloproteinases-2 (MMP-2), tissue inhibitor of metalloproteinase-1 (TIMP-1), tissue inhibitor of metalloproteinase-2 (TIMP-2), and cathepsin B were analyzed by Western blotting in the LN229 (**A**) and U87MG cells (**B**) using the shScramble control group (004 and 003) and shFABP6. GAPDH was used as the loading control. The plotted graphs show the relative quantitative analysis of the aforementioned proteins. * *p* < 0.05; ** *p* < 0.01; *** *p* < 0.001 compared with the shScramble control group.

The invasion-related proteins were investigated after the attenuation of FABP6. After the knockdown of FABP6, there was a significant decrease in the expression levels of active MMP-2 and cathepsin B (Figure 4). In addition, the levels of the tissue inhibitors of metalloproteinases (TIMP)-1 and TIMP-2 decreased after FABP6 reduction in glioma cells (Figure 4). These results implied that the decrease in the migration and invasion abilities after FABP6 knockdown may be due to the reductions in the levels of MMPs and cathepsin B and the enhancement of the TIMPs in glioma cells.

### 3.4. Tube Formation of Endothelial Cells Was Attenuated by the Knockdown of FABP6 in Glioma Cells

**Figure 5:**
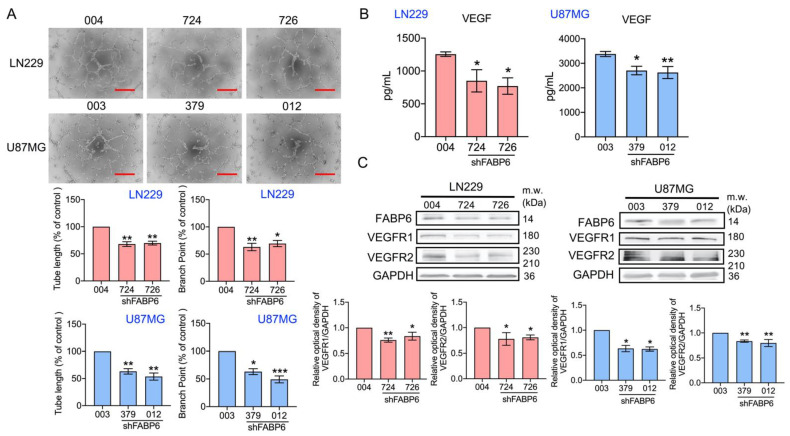
The effect of the knockdown of FABP6 on angiogenesis. The human umbilical vein endothelial cells (HUVECs) were cultured with an FABP6 knockdown conditioned medium (CM) or scramble control CM of glioma cells for 6 h. (**A**) The formation of an endothelial cell network was observed and the number of branch points and tube length in the LN229 and U87MG CM were analyzed. The plotted graphs show the relative attenuation of branch points and tube lengths in the FABP6 knockdown CM compared with those in the scramble control group. Scale bar = 200 μm. (**B**) The concentration levels of vascular endothelial growth factors (VEGFs) in the CM of LN229 and U87MG cells were analyzed by the enzyme-linked immunosorbent assay (ELISA). (**C**) The expression levels of the vascular endothelial growth factor receptor 1 (VEGFR1) and vascular endothelial growth factor receptor 2 (VEGFR2) were analyzed by Western blotting in the LN229 and U87MG cells using the shScramble control group and shFABP6. GAPDH was used as the loading control. The plotted graphs show the relative quantitative analysis of the aforementioned proteins. * *p* < 0.05; ** *p* < 0.01; *** *p* < 0.001 compared with the shScramble control group.

Next, the endothelial network formation was analyzed after FABP6 knockdown in glioma cells. As shown in Figure 5A, angiogenesis ability, including branch points and tube length, was reduced in the shFABP6 groups. Furthermore, the expression levels of VEGF in the glioma conditioned medium (CM) were examined. The concentration of VEGF in CM decreased significantly after FABP6 inhibition compared to that in the scramble control (Figure 5B). Moreover, the expression levels of VEGFR1 and VEGFR2 were significantly reduced after FABP6 inhibition in glioma cells (Figure 5C). The addition of VEGFA reversed the tube formation ability in shFABP6 group (Figure S3). These results indicated that the angiogenic ability of endothelial cells was decreased by FABP6 knockdown in glioma cells, which might be due to the decrease in VEGF secretion in glioma cells.

### 3.5. The Reduced Phosphorylation of JNK, ERK, and p65 Was Caused by FABP6 Inhibition

**Figure 6:**
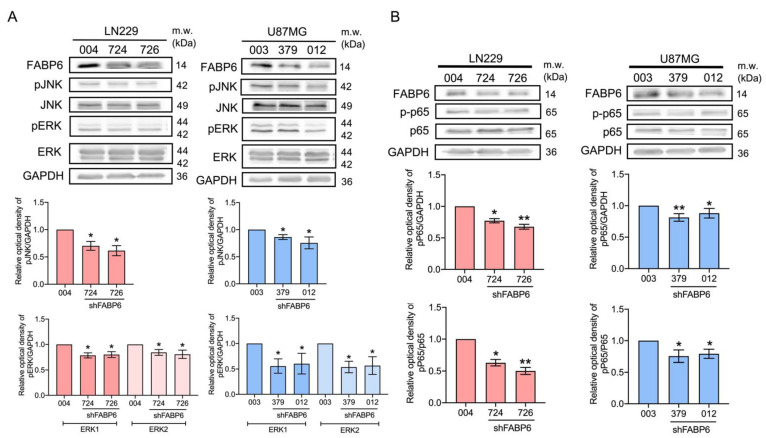
Effects of c-Jun NH2-terminal kinase (JNK), extracellular signal-regulated kinase (ERK), and p65 after FABP6 knockdown. The expression levels of phospho-c-Jun NH2-terminal kinase (p-JNK), JNK, phospho-extracellular signal-regulated kinase (p-ERK), ERK (**A**), phospho-p65 (p-p65), and p65 (**B**) were analyzed by Western blotting in the LN229 and U87MG cells using the shScramble control group and shFABP6. GAPDH was used as the loading control. The plotted graphs show the relative quantitative analysis of the aforementioned proteins. * *p* < 0.05 and ** *p* < 0.01 compared with the shScramble control group.

As the phosphorylation of extracellular signal-regulated kinase (ERK) and c-Jun NH2-terminal kinase (JNK) promotes glioma cell invasion and migration [12], we examined the p-ERK and p-JNK expression after FABP6 knockdown. In both the LN229 and U87MG cell lines, p-JNK and p-ERK decreased with FABP6 attenuation (Figure 6A). NF-κB is activated in malignant glioma and anti-p65 antibodies inhibit invasion and angiogenesis in glioma cells [13,14]. The p-p65 expression decreased significantly after FABP6 inhibition in the LN229 and U87MG cells (Figure 6B). The decreased expression levels of p-JNK, p-ERK, and p-p65 may attenuate invasion and angiogenesis in glioma cells.

### 3.6. FABP6 Knockdown Combined with TMZ Application Attenuated Tumor Progression in the Orthotropic Xenograft Model

**Figure 7:**
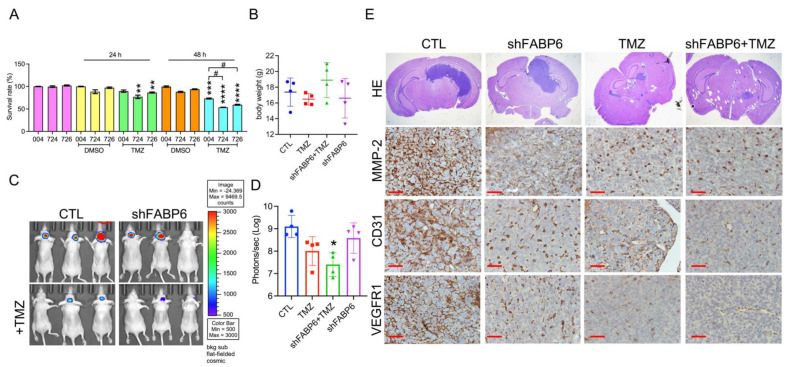
The effect of FABP6 knockdown on the tumor progression of LN229 cells in an orthotropic xenograft mouse model: (**A**) the survival rates of the glioma cell line in scrambled cytotoxic T lymphocyte (CTL) and shFABP6 combined with or without 100 μM temozolomide (TMZ) for 24 and 48 h (*n* = 6); (**B**) the body weights of animals are shown; (**C**) the effects of different groups on the size and growth of tumors were observed using bioluminescent imaging with an in vivo imaging system (IVIS); (**D**) quantification of the bioluminescence data in different groups; (**E**) HE staining of the xenograft orthotropic brain tissues. * *p* < 0.05; ** *p* < 0.01; *** *p* < 0.001; **** *p* < 0.0001 compared with the DMSO control group. # *p* < 0.05 compared with shScramble control + TMZ group. Scale bar = 50 μm.

FABP6 reduction did not affect the survival rate of LN229 glioma cells compared to that of control cells. When FABP6 knockdown combined with 100 μM TMZ for 48 h, the survival rate declined significantly compared with the CTL + TMZ group, indicating a synergistic effect of FABP6 inhibition and TMZ application (Figure 7A). An orthotropic xenograft mouse model was then used to verify the in vitro findings. The average body weight did not differ between the different groups (Figure 7B). After 15 days, the bioluminescence images were captured using an IVIS system (Figure 7C). When FABP6 knockdown was combined with TMZ treatment, the tumor regressed significantly compared to the CTL group (Figure 7D). Moreover, the expression of MMP-2, CD31, and VEGFR1 decreased in the FABP6 knockdown combined with the TMZ group (Figure 7E). In addition, FABP6 inhibition combined with TMZ induced more apoptotic cells than those in the control group by TUNEL staining (Figure S4). These results indicated that FABP6 knockdown combined with TMZ attenuates tumor progression in animal models.

## 4. Discussion

A FABP6 has been reported to be a cancer-related protein in colorectal cancer [9,15]. According to our results, the expression of FABP6 in gliomas was higher than normal tissue. Therefore, further investigation of the role of FABP6 in malignant glioma is imperative. However, most studies of fatty acid binding protein in glioma were limited to FABP7, which was found to be overexpressed and thought to be involved in tumor proliferation, invasion, and migration [10]. The significance of FABP6 attracts researcher’s interest as a regulator of cholesterol metabolism in gastrointestinal cancer [15]. Recently, targeting a cholesterol pathway has been proposed in anti-glioblastoma therapy [16]. Elsherbiny et al. reveals that these glioma properties might be related to fatty acid metabolism [17]. Therefore, the knockdown of FABP6 blocked migration, invasion, and angiogenesis, which may be associated with bile acid metabolism.

The attenuation of FABP6 inhibited the migration of malignant glioma cells. Herein, we analyzed the migration-related proteins to determine the impact of FABP on tumor migration. It is known that the regulation of MLC affects cell migration [18]. Further, the expression of phosphorylated MLC and ERK decreased after knocking down FABP6. This implies that tumor migration might be enhanced via ERK and myosin light-chain phosphorylation pathways. In breast cancer, the activation of myosin light-chain kinase (MLCK) leads to MLC phosphorylation, which promotes cancer cell migration via the ERK signaling pathway [19]. Notably, even the expression levels of phosphorylated FAK and paxillin, which are associated with cell motility and adhesion [20], increased after FABP6 knockdown. Further, the migration ability of the neoplasm was still inhibited. Such finding indicated that phosphorylation of paxillin may inhibit cell motility, which is similar to another study that investigated the role of phosphorylated paxillin in normal murine mammary gland epithelial cells [21]. These contradictory results indicate that FABP6 may regulate cell migration through other signaling molecules or cascades, and further research is required.

As mentioned earlier, the expression of MMP-2 and cathepsin B was significantly reduced after FABP6 knockdown in our study. Conversely, TIMP-1 expression was increased. These proteins are known to be associated with tumor invasion. MMPs mediate the breakdown of the basal membrane, degrade the extracellular matrix, and create a microenvironment that enhances tumor cell survival [22]. On the other hand, TIMPs serve as inhibitors of MMPs. In oral squamous cell carcinoma, FABP5 regulates MMP-9 expression and tumor invasion [23]. Overexpression of FABP4 in prostate cancer results in the upregulation of MMP-2 and MMP-9, which promotes the invasion of cancer cells [7]. In a cerebral ischemia injury model, FABP4 promotes MMP-9 expression through JNK/c-Jun signaling [24]. In colon cancer, FABP4 enhances epithelial–mesenchymal transition and the associated proteins, including MMP-2, MMP-9, and E-cadherin via the AKT pathway [25]. Cathepsin B, which plays a role in neoplastic invasiveness and neovascularization, can be activated by NF-kB in osteosarcoma [26]. In addition, inhibition of NF-kB in glioma cells decreases MMP-9 and VEGF expression, leading to invasion and angiogenesis blockade [14]. In our study, p-JNK and p-p65 were decreased as FABP6 was reduced, which may inhibit MMP-2 and cathepsin B expression in glioma. Collectively, the reduction in p-ERK, p-JNK, and p-65 impaired tumor progression in FABP6 inhibition.

The combination of FABP6 inhibition and TMZ reduced cell survive in LN229 cells (Figure 7A). Perazzoli et al. has been reported that TMZ treatment induced cell cycle arrest at G2/M phase in LN229 cells [27]. In addition, by causing DNA damage, TMZ treatment leads to apoptosis in LN229 cells [28]. The possible combination effect on cell survival reduction may be due to the apoptosis by cleaved PARP expression (data not shown) and TUNEL positive cells in FABP6 knockdown combined with TMZ group (Figure S4). These results suggest that FABP6 inhibition may enhance the sensitivity of TMZ treatment in GBM cells.

Conventional GBM therapies lack potent drugs targeting the lipid metabolism pathway [16]. Our results suggest targeting FABP6 combined with TMZ can ameliorate glioma proliferation and migration. Pharmacological agents to modify FABP function has been proposed with tissue-specific or cell-type-specific control of lipid pathways [29]. In addition, targeting cholesterol synthesis in brain tumors might render their proliferation without compromising cell viability in other organs [16]. However, only inhibitors for FABP4 are available for research [29]. Animal studies reveal ideal response to FABP4 inhibitors in asthma, obesity, and type 2 diabetes mellitus without significant toxicity [30,31]. For targeting FABP6 in glioma patient therapy, specific FABP6 inhibitors or research into other modulators of the FABP6 pathway is recommended.

After knockdown of FABP6, angiogenesis was attenuated in HUVECs, accompanied by the decreased expression levels in VEGF in the CM and VEGFRs in glioma cells. A previous study indicated that FABP5 promotes tumor angiogenesis and activates the VEGF-A pathway in HCC via the peroxisome proliferator-activated receptor d (PPARd)-dependent pathway [32]. In addition, liver FABP1 interacts with VEGFR2 in HCC, further activating specific pathways, and results in VEGF-A upregulation, thereby promoting angiogenesis and tumor migration [33]. Moreover, FABP4 has been reported to serve as a target of the VEGF/VEGFR2 signaling pathway in endothelial cells and affects vascular sprouting in ovarian cancer [34]. Taken together, the results of this study indicate that FABP6 may interact with the VEGF/VEGFR signaling pathway to control angiogenesis in gliomas; however, this needs to be investigated in future studies.

## 5. Conclusions

This is the first study to investigate the role of FABP6 in malignant glioma. In our study, the knockdown of FABP6 resulted in the inhibition of the invasion, migration, and angiogenesis in glioma. These findings indicated that FABP6 may serve as a potential target for therapeutic strategies in gliomas in the future.

## Figures and Tables

**Table 1:** Information on the antibodies.

Name	Species	Brand	Catalog Number
Cathepsin B	Rb	Abcam	ab125067
CD31	Rb	Abcam	ab28364
ERK	Rb	Cell Signaling Technology	9102s
FABP6	Rb	Novus	NBP1-32482
FAK	Rb	Cell Signaling Technology	71433s
GAPDH	Rb	Cell Signaling Technology	5174s
JNK	Rb	Cell Signaling Technology	9252s
MLC 2	Rb	Cell Signaling Technology	8505s
MMP2	Rb	Cell Signaling Technology	13132s
MMP9	Rb	Cell Signaling Technology	13667s
P65	Rb	Cell Signaling Technology	8242s
Paxillin	Ms	BD Biosciences	610051
p-ERK	Rb	Cell Signaling Technology	4377s
p-FAK (Y397)	Ms	BD Biosciences	611723
p-JNK	Rb	Cell Signaling Technology	4668s
p-MLC (Ser19)	Rb	Cell Signaling Technology	3671
p-p65	Rb	Abcam	Ab185619
p-paxillin	Rb	Cell Signaling Technology	2541s
TIMP-1	Rb	Abcam	ab109125
TIMP-2	Ms	Millipore	MAB13446
VEGFR1	Rb	Abcam	ab32152
VEGFR2	Rb	Cell Signaling Technology	2479s

Rb and Ms indicated anti-rabbit and anti-mouse, respectively.

## Data Availability

The data presented in this study are available on request from the corresponding author.

## Supplementary Materials

The following are available online at https://www.mdpi.com/article/10.3390/cells10102782/s1, Figure S1: The correlation of FABP6 expression and survival time; Figure S2: Establishment of the fatty acid-binding protein 6 (FABP6)-attenuated glioma cell lines; Figure S3: The effect of VEGFA application on tube formation in FABP6 knockdown LN229 cells; Figure S4: TUNEL staining of the xenograft orthotropic brain tissues.

## References

[1] Ostrom Q.T., Bauchet L., Davis F.G., Deltour I., Fisher J.L., Langer C.E., Pekmezci M., Schwartzbaum J.A., Turner M.C., Walsh K.M. (2014). The epidemiology of glioma in adults: A “state of the science” review. Neuro-Oncology.

[2] Louis D.N., Perry A., Reifenberger G., von Deimling A., Figarella-Branger D., Cavenee W.K., Ohgaki H., Wiestler O.D., Kleihues P., Ellison D.W. (2016). The 2016 World Health Organization Classification of Tumors of the Central Nervous System: A summary. Acta Neuropathol..

[3] Das S., Marsden P.A. (2013). Angiogenesis in glioblastoma. N. Engl. J. Med..

[4] Jing C., Beesley C., Foster C.S., Rudland P.S., Fujii H., Ono T., Chen H., Smith P.H., Ke Y. (2000). Identification of the messenger RNA for human cutaneous fatty acid-binding protein as a metastasis inducer. Cancer Res..

[5] Tolle A., Suhail S., Jung M., Jung K., Stephan C. (2011). Fatty acid binding proteins (FABPs) in prostate, bladder and kidney cancer cell lines and the use of IL-FABP as survival predictor in patients with renal cell carcinoma. BMC Cancer.

[6] Amiri M., Yousefnia S., Seyed Forootan F., Peymani M., Ghaedi K., Nasr Esfahani M.H. (2018). Diverse roles of fatty acid binding proteins (FABPs) in development and pathogenesis of cancers. Gene.

[7] Huang M., Narita S., Inoue T., Koizumi A., Saito M., Tsuruta H., Numakura K., Satoh S., Nanjo H., Sasaki T. (2017). Fatty acid binding protein 4 enhances prostate cancer progression by upregulating matrix metalloproteinases and stromal cell cytokine production. Oncotarget.

[8] Elmasri H., Ghelfi E., Yu C.W., Traphagen S., Cernadas M., Cao H., Shi G.P., Plutzky J., Sahin M., Hotamisligil G. (2012). Endothelial cell-fatty acid binding protein 4 promotes angiogenesis: Role of stem cell factor/c-kit pathway. Angiogenesis.

[9] Ohmachi T., Inoue H., Mimori K., Tanaka F., Sasaki A., Kanda T., Fujii H., Yanaga K., Mori M. (2006). Fatty acid binding protein 6 is overexpressed in colorectal cancer. Clin. Cancer Res..

[10] De Rosa A., Pellegatta S., Rossi M., Tunici P., Magnoni L., Speranza M.C., Malusa F., Miragliotta V., Mori E., Finocchiaro G. (2012). A radial glia gene marker, fatty acid binding protein 7 (FABP7), is involved in proliferation and invasion of glioblastoma cells. PLoS ONE.

[11] Martin D.D., Robbins M.E., Spector A.A., Wen B.C., Hussey D.H. (1996). The fatty acid composition of human gliomas differs from that found in nonmalignant brain tissue. Lipids.

[12] McCubrey J.A., Steelman L.S., Chappell W.H., Abrams S.L., Wong E.W., Chang F., Lehmann B., Terrian D.M., Milella M., Tafuri A. (2007). Roles of the Raf/MEK/ERK pathway in cell growth, malignant transformation and drug resistance. Biochim. Biophys. Acta.

[13] Wang H., Wang H., Zhang W., Huang H.J., Liao W.S., Fuller G.N. (2004). Analysis of the activation status of Akt, NFkappaB, and Stat3 in human diffuse gliomas. Lab. Investig..

[14] Puliyappadamba V.T., Hatanpaa K.J., Chakraborty S., Habib A.A. (2014). The role of NF-kappaB in the pathogenesis of glioma. Mol. Cell Oncol..

[15] Zhang Y., Zhao X., Deng L., Li X., Wang G., Li Y., Chen M. (2019). High expression of FABP4 and FABP6 in patients with colorectal cancer. World J. Surg. Oncol..

[16] Ahmad F., Sun Q., Patel D., Stommel J.M. (2019). Cholesterol metabolism: A potential therapeutic target in glioblastoma. Cancers.

[17] Elsherbiny M.E., Emara M., Godbout R. (2013). Interaction of brain fatty acid-binding protein with the polyunsaturated fatty acid environment as a potential determinant of poor prognosis in malignant glioma. Prog. Lipid Res..

[18] Totsukawa G., Wu Y., Sasaki Y., Hartshorne D.J., Yamakita Y., Yamashiro S., Matsumura F. (2004). Distinct roles of MLCK and ROCK in the regulation of membrane protrusions and focal adhesion dynamics during cell migration of fibroblasts. J. Cell Biol..

[19] Zhou X., Liu Y., You J., Zhang H., Zhang X., Ye L. (2008). Myosin light-chain kinase contributes to the proliferation and migration of breast cancer cells through cross-talk with activated ERK1/2. Cancer Lett..

[20] Lopez-Colome A.M., Lee-Rivera I., Benavides-Hidalgo R., Lopez E. (2017). Paxillin: A crossroad in pathological cell migration. J. Hematol. Oncol..

[21] Yano H., Uchida H., Iwasaki T., Mukai M., Akedo H., Nakamura K., Hashimoto S., Sabe H. (2000). Paxillin alpha and Crk-associated substrate exert opposing effects on cell migration and contact inhibition of growth through tyrosine phosphorylation. Proc. Natl. Acad. Sci. USA.

[22] Deryugina E.I., Quigley J.P. (2015). Tumor angiogenesis: MMP-mediated induction of intravasation- and metastasis-sustaining neovasculature. Matrix. Biol..

[23] Fang L.Y., Wong T.Y., Chiang W.F., Chen Y.L. (2010). Fatty-acid-binding protein 5 promotes cell proliferation and invasion in oral squamous cell carcinoma. J. Oral. Pathol. Med..

[24] Liao B., Geng L., Zhang F., Shu L., Wei L., Yeung P.K.K., Lam K.S.L., Chung S.K., Chang J., Vanhoutte P.M. (2020). Adipocyte fatty acid-binding protein exacerbates cerebral ischaemia injury by disrupting the blood-brain barrier. Eur. Heart J..

[25] Tian W., Zhang W., Zhang Y., Zhu T., Hua Y., Li H., Zhang Q., Xia M. (2020). FABP4 promotes invasion and metastasis of colon cancer by regulating fatty acid transport. Cancer Cell Int..

[26] Hamer I., Delaive E., Dieu M., Abdel-Sater F., Mercy L., Jadot M., Arnould T. (2009). Up-regulation of cathepsin B expression and enhanced secretion in mitochondrial DNA-depleted osteosarcoma cells. Biol. Cell.

[27] Perazzoli G., Prados J., Ortiz R., Caba O., Cabeza L., Berdasco M., Gonzalez B., Melguizo C. (2015). Temozolomide Resistance in Glioblastoma Cell Lines: Implication of MGMT, MMR, P-Glycoprotein and CD133 Expression. PLoS ONE.

[28] Hermisson M., Klumpp A., Wick W., Wischhusen J., Nagel G., Roos W., Kaina B., Weller M. (2006). O6-methylguanine DNA methyltransferase and p53 status predict temozolomide sensitivity in human malignant glioma cells. J. Neurochem..

[29] Furuhashi M., Hotamisligil G.S. (2008). Fatty acid-binding proteins: Role in metabolic diseases and potential as drug targets. Nat. Rev. Drug Discov..

[30] Furuhashi M., Tuncman G., Görgün C.Z., Makowski L., Atsumi G., Vaillancourt E., Kono K., Babaev V.R., Fazio S., Linton M.F. (2007). Treatment of diabetes and atherosclerosis by inhibiting fatty-acid-binding protein aP2. Nature.

[31] Sulsky R., Magnin D.R., Huang Y., Simpkins L., Taunk P., Patel M., Zhu Y., Stouch T.R., Bassolino-Klimas D., Parker R. (2007). Potent and selective biphenyl azole inhibitors of adipocyte fatty acid binding protein (aFABP). Bioorganic Med. Chem. Lett..

[32] Pan L., Xiao H., Liao R., Chen Q., Peng C., Zhang Y., Mu T., Wu Z. (2018). Fatty acid binding protein 5 promotes tumor angiogenesis and activates the IL6/STAT3/VEGFA pathway in hepatocellular carcinoma. Biomed. Pharm..

[33] Ku C.Y., Liu Y.H., Lin H.Y., Lu S.C., Lin J.Y. (2016). Liver fatty acid-binding protein (L-FABP) promotes cellular angiogenesis and migration in hepatocellular carcinoma. Oncotarget.

[34] Harjes U., Bridges E., Gharpure K.M., Roxanis I., Sheldon H., Miranda F., Mangala L.S., Pradeep S., Lopez-Berestein G., Ahmed A. (2017). Antiangiogenic and tumour inhibitory effects of downregulating tumour endothelial FABP4. Oncogene.

