# Supplementary material for: Inhibition of FABP6 Reduces Tumor Cell Invasion and Angiogenesis through the Decrease in MMP-2 and VEGF in Human Glioblastoma Cells
Source: Cells. 2021 Oct 17;10(10):2782. doi: 10.3390/cells10102782 (PMC8534568; doi:10.3390/cells10102782)
Supplement: Supplementary file 1 [file cells-10-02782-s001.zip › cells-1390316-supplementary.pdf]

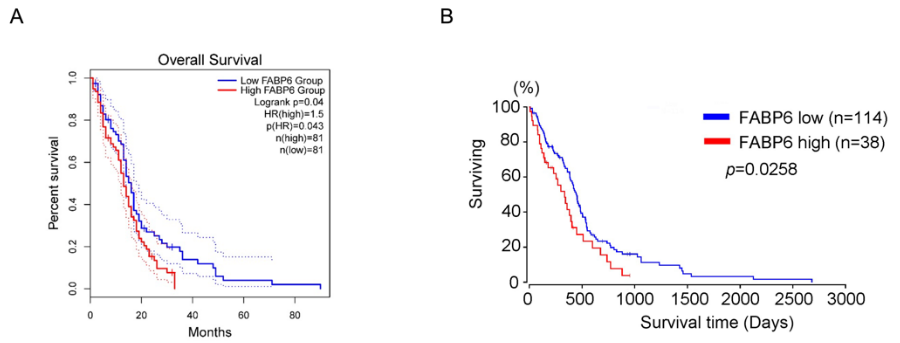

Supplementary Figure S1 The correlation of FABP6 expression and survival time. The analysis of FABP6 and overall survival using (A) the Gene Expression Profiling Interactive Analysis (GEPIA) website and (B) The Cancer Genome Atlas (TCGA) database.

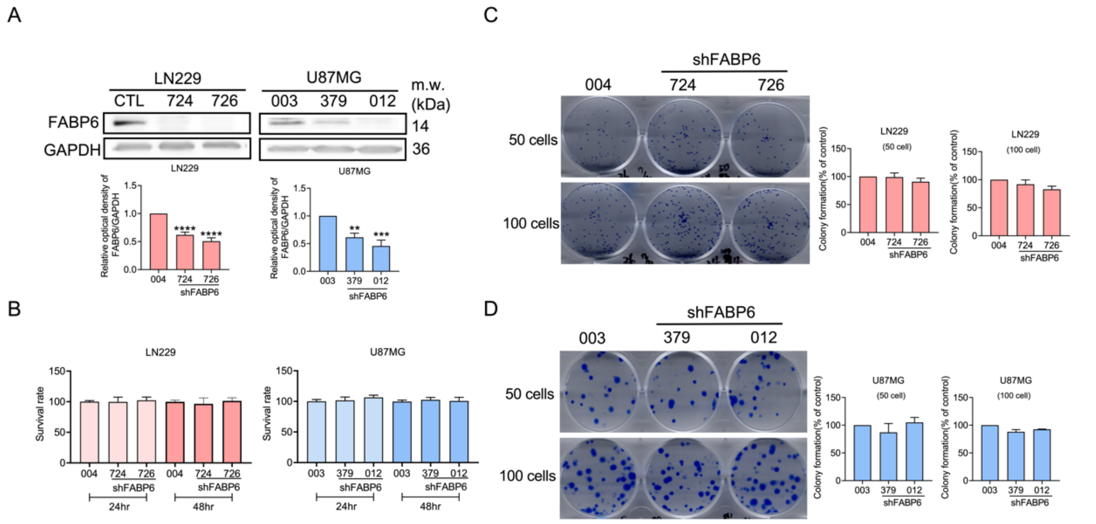

Supplementary Figure S2 Establishment of the fatty acid-binding protein 6 (FABP6)-attenuated glioma cell lines. (A) The protein expression levels of FABP6 in the LN229 and U87MG cells after shFABP6 knockdown. * $P < 0.05$; ** $P < 0.01$; *** $P < 0.001$; **** $P < 0.0001$ compared with the shScramble control group (004 in LN229 cells and 003 in U87MG cells). (B) The 3-(4, 5-dimethylthiazol-2-yl)-2, 5-diphenyltetrazolium bromide (MTT) assay was performed to evaluate the cell survival rates of the LN229 and U87MG cells at 24 h and 48 h after seeding. (C, D) Colony formation was achieved by culturing 50 cells and 100 cells to demonstrate the long-term effects of cell growth in LN229 and U87MG cells.

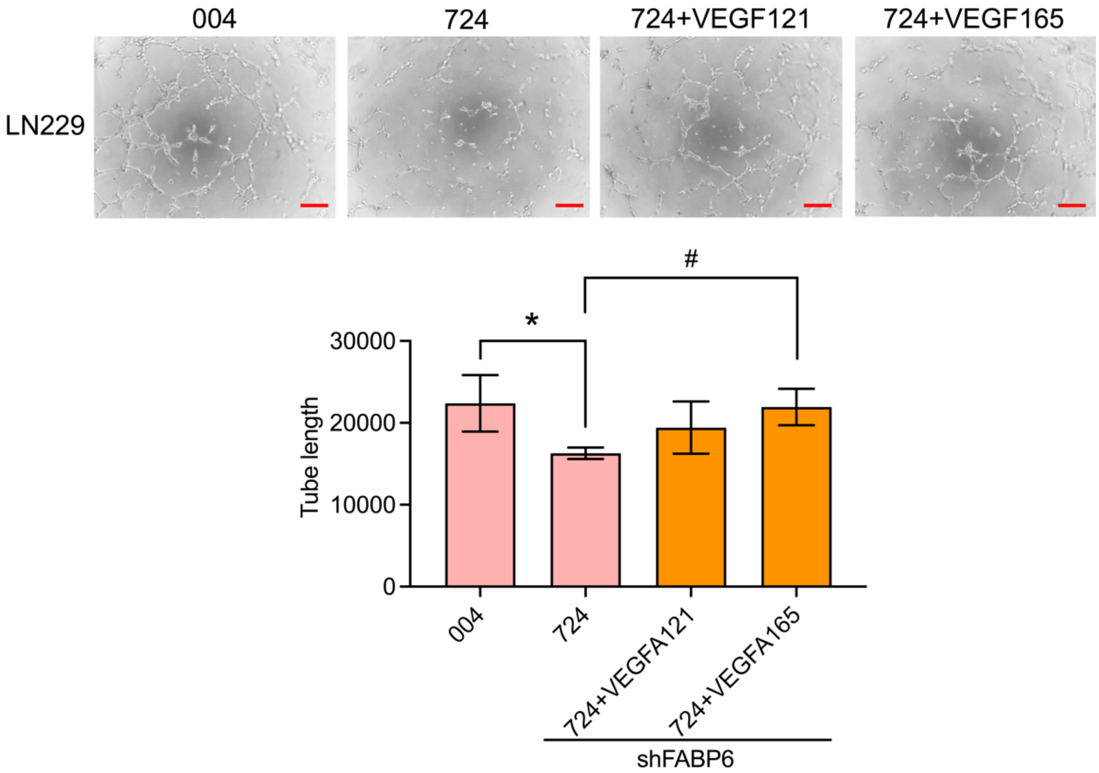

Supplementary Figure S3 The effect of VEGFA application on tube formation in FABP6 knockdown LN229 cells. Conditioned medium was collected after 48 h cell culture incubation. Tube formation assay was performed with the addition of VEGFA121 (500 pg/mL) or VEGFA165 (500 pg/mL) in shFABP6 group (724). Total length of HUVECs was captured and measured after 6 h incubation. * $P < 0.05$; # $P < 0.05$ compared with the shScramble control group (004) and shFABP6 group (724). Scale bar = 200 μm.

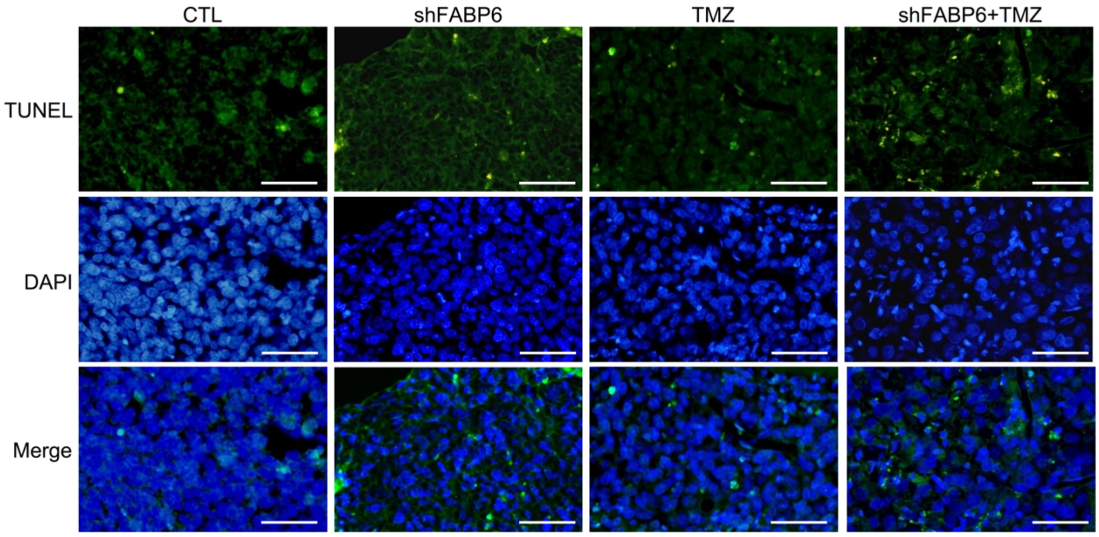

Supplementary Figure S4 TUNEL staining of the xenograft orthotropic brain tissues. DAPI showed the nucleus. Scale bar = 25 μm.
